# California’s Supplemental Nutrition Assistance Program-Education (SNAP-Ed) *Be Better* social marketing campaign: mothers’ fruit and vegetable consumption and facilitation of children’s healthy behaviours

**DOI:** 10.1017/S1368980023001301

**Published:** 2023-11

**Authors:** Ingrid M Cordon, Celeste Doerr, Lauren Whetstone

**Affiliations:** 1 California Department of Public Health, Nutrition Education and Obesity Prevention Branch, 1616 Capitol Ave, Sacramento, CA 95814, USA; 2 California Department of Public Health, Maternal, Child and Adolescent Health Division, 1615 Capitol Ave, Sacramento, CA 95814, USA

**Keywords:** Social marketing, SNAP-Ed, Fruits and vegetables, Water, California

## Abstract

**Objective:**

Evaluation of California Department of Public Health’s three-year social marketing campaign (*Be Better*) to encourage healthy eating and water consumption among Supplemental Nutrition Assistance Program-Education (SNAP-Ed) California mothers. Andreasen’s social marketing framework was used to outline the development and evaluation of the campaign.

**Design:**

Quantitative, pre-post cross-sectional study with three cohorts nested within survey years. Generalised estimating equation modeling was used to obtain population estimates of campaign reach and changes in mothers’ fruit and vegetable (FV) consumption and facilitative actions towards their children’s health behaviours.

**Setting:**

*CalFresh* Healthy Living (California’s SNAP-Ed).

**Participants:**

Three separate cohorts of SNAP mothers were surveyed (pre, post) between 2016 and 2018 inclusive. A total of 2229 mothers (ages 18–59) self-identified as White, Latina, African American or Asian/Pacific Islander participated.

**Results:**

Approximately 82 percent of surveyed mothers were aware of the campaign as assessed by measures of recall and recognition. Ad awareness was positively associated with mothers’ FV consumption (R^2^ = 0·45), with the proportion of FV on plates and with behaviours that facilitate children’s FV consumption and limit unhealthy snacks and sugary drinks (*βs* ranged from 0·1 to 0·7).

**Conclusions:**

The campaign successfully reached 82 percent of surveyed mothers. Positive associations between California’s *Be Better* campaign and targeted health behaviours were observed, although the associations varied by year and media channel (i.e. television, radio, billboards and digital). Most associations between ad awareness and outcomes were noted in years two and three of the campaign, suggesting that more than 1 year of campaign exposure was necessary for associations to emerge.

Although associations between lifestyle behaviours and overall health are complex, there is substantial evidence that a healthy lifestyle (e.g. eating healthy and drinking water) can help people maintain good health and reduce the risk of chronic diseases^(1)^. Eating patterns in the United States, however, remain far below the United States Department of Agriculture’s (USDA) dietary guidelines^(2)^. Only 12 percent of adults in 2019 met the USDA’s fruit intake recommendation and only 10 percent met the vegetable recommendation^(3–5)^. Moreover, while current USDA guidelines recommend keeping added sugars (typically found in processed foods) to less than 10 percent of daily calorie intake, the typical U.S. diet includes more than 13 percent, with sugar-sweetened beverages (SSB) accounting for approximately 35 percent of those added sugars^(2)^. Poor dietary quality, such as inadequate fruit and vegetable (FV) intake, has been linked to weight gain, obesity and obesity-related diseases that pose significant risks to public health^(6,7)^.

There are concerns that healthy eating patterns are complicated by disparities in income, with adults and children with low incomes eating fewer FV and consuming more SSB than families with higher incomes. As incomes go up, the percentage of adults eating vegetables and the frequency of fruit intake also increases^(3,4)^. Research indicates that individuals who receive Supplemental Nutrition Assistance Program (SNAP) benefits (a USDA nutrition assistance program for families with low incomes) have poorer quality diets and consume fewer FV than individuals with higher incomes overall^(8,9)^. SNAP recipients are also more likely to report higher levels of SSB consumption compared with non-recipients^(10)^. Income-related disparities can also be seen in obesity rates^(11)^. The overall U.S. obesity prevalence (defined as a Body Mass Index, or BMI, > 29·9) is estimated at 41·9 percent (2017–2020)^(12)^. When examined by income, those in the lowest income-to-poverty ratio (≤ 130 %) had a prevalence of 43·9 percent, whereas those in the highest income-to-poverty ratio (> 350 %) had a prevalence of 39·0 percent^(12)^.

The California Department of Public Health’s (CDPH) SNAP-Education (SNAP-Ed) program (a USDA nutrition education and obesity prevention program) strives to improve the health of Californians with low incomes through comprehensive public health approaches to prevent obesity. Like the overall adult patterns in the USA, only 14 percent of California adults met the fruit recommendation in 2019 and only 11 percent met the vegetable recommendation^(13)^. Among those in the lowest income-to-poverty ratio, only 9 percent of California adults met the vegetable recommendation compared to 13 percent in the highest income-to-poverty ratio^(13)^. Furthermore, in 2017, SNAP-Ed eligible California adults reported drinking more SSB on average than adults in higher income households^(14)^. In 2021, the estimated obesity rate among SNAP-Ed eligible California adults was 46·8 percent compared with 36·9 percent among adults from higher income households^(15)^. Thus, there is a great need for programs and interventions that can assist SNAP-Ed eligible Californians achieve healthier behaviours and reduce the risk of obesity and its associated chronic diseases.

Through the California Department of Social Services, CDPH provides funding for training and technical assistance to local health departments and for state-wide, community-wide and individual education interventions. Complementing these efforts is a yearly social marketing campaign that encourages Californians with low incomes to lead healthier lives. One such campaign was the *Be Better* social marketing campaign that aired between 2016 and 2018 with SNAP-Ed households as the priority population.

Here, we describe the development of the *Be Better* campaign and its evaluation using Andreasen’s social marketing benchmark criteria^(16)^. For the evaluation, we examined maternal FV consumption (frequency and proportions) and, because mothers significantly influence their children’s behaviours, we also examined maternal actions that facilitate children’s healthy behaviours^(17)^. Although the campaign included three messages (nutrition, water and physical activity), the present paper focuses on nutrition and water messages and will not address physical activity. We begin with a brief outline of the social marketing framework and then describe the formative research used to develop the campaign using Andreasen’s benchmark criteria. Finally, we outline the campaign evaluation and its results.

## Social marketing and Andreasen’s social marketing framework

As defined by the International, European and Australian Social Marketing Associations, social marketing ‘seeks to develop and integrate marketing concepts with other approaches to influence behaviours that benefit individuals and communities for the greater social good’^(18)^. A distinguishing feature of social marketing is the concept of exchanges (Exchange Theory) – a subjective cost–benefit analysis – that postulates people will assume a new behaviour in exchange for benefits that outweigh the costs of a current behaviour^(19)^. A core element of social marketing, therefore, is the creation of attractive exchanges (e.g. messages) that encourage behaviour change^(16)^.

Andreasen outlines six benchmark criteria (audience research, segmentation, exchange, behaviour change, competition and marketing mix) in the development of an effective social marketing campaign^(16)^. Research suggests that the more benchmarks are included in the development of a campaign, the more likely the campaign will be effective^(20–23)^. Key elements include the techniques used to identify the groups of individuals for whom messages are developed that provide the framework for understanding the needs, barriers and behavioural facilitators specific to the target audience^(20)^. We consider Andreasen’s benchmarks, in relation to the *Be Better* campaign, in the following sections.

## Audience research: development of the *Be Better* social marketing campaign

Audience research is necessary to better understand the characteristics, beliefs, motivations and behaviours of a target audience^(16,20)^. As part of this process, a formative study was conducted to better understand CDPH’s primary audience – SNAP-Ed families.

The *Be Better* campaign (created by Runyon Saltzman and Einhorn Inc. and later modified by Rescue Agency) was designed to inspire and empower mothers with low incomes to make healthy lifestyle changes. Using the Integrative Model of Behavior Change as a theoretical framework^(24)^, the formative research study determined that a *social normative* approach had the strongest potential for changing the health behaviours of California mothers with low incomes^(25)^.

Normative social influence refers to ‘a conformity to group norms brought on by a desire to be liked by group members’^(26)^. It suggests that seeing others engaged in an action (even indirectly) can have a powerful effect on behaviour^(26)^. The social normative message of the *Be Better* campaign implied that we could build healthier families and communities and that many parents were already working towards that end.

The formative study found that the campaign’s social normative messaging had a positive influence on self-efficacy beliefs – beliefs about one’s ability to perform a desired behaviour^(25)^. Self-efficacy beliefs have been identified as an important contributor to healthy behaviours^(27)^. The *Be Better* campaign sought to increase self-efficacy beliefs around healthy behaviours through messaging that focused on positive social norms. Campaign ads showed mothers and families making small behavioural changes that could help them feel better and healthier. The campaign integrated these messages with tools (e.g. tips and recipes) and resources (a dedicated website) that provided more in-depth information to help mothers build healthy skills and increase self-efficacy.

The formative research also found that mothers were generally driven and motivated by a desire to spend quality time with family. Participants wanted to connect with, and hear from, other moms who were similarly motivated to make healthy changes. Mothers were interested in pursuing activities that would create memories and strengthen family bonds while using strategies that were presented in simple and approachable ways – observations noted in other SNAP-Ed formative work^(28–30)^. Stress relief, health benefits and feeling and looking better were strong motivators to individual change.

Following the formative research, focus groups were conducted with SNAP-Ed eligible California mothers to test the *Be Better* messages, ads and marketing materials. In general, mothers indicated that the campaign was relatable, realistic, believable, emotionally compelling and motivating. Phrases that were perceived as vague or too complex by mothers were not incorporated into the campaign. When probed regarding feelings of self-efficacy to perform the ad suggestions, mothers indicated the ads made them feel that they could and wanted to perform the behaviours promoted in the ads, such as engaging in healthy activities with their family. Mothers also felt that the ads clearly communicated the message that taking small steps could improve their general health.

## Audience segmentation

Segmentation is key to identifying the different groups (or different segments) of individuals for whom a unique campaign is designed^(16,31)^. Considering California’s diverse population and the obesity rates among these groups, the primary audiences selected for the *Be Better* campaign were SNAP-Ed eligible English and Spanish language women with school-aged children. The BMI among California women (all races) who met SNAP-Ed eligibility in 2021, for example, was estimated to be 35·2 percent compared with 26·5 percent for women with incomes above SNAP-Ed eligibility^(15)^. These selected groups comprise a large portion of the California SNAP-Ed population, with incomes at or below 185 percent of the federal poverty level (an economic measure used in the U.S. to determine whether a family’s household size and income qualifies them for federal programs). The formative research study suggested that the social normative approach would appeal to SNAP-Ed mothers and had a strong potential for changing health behaviours.

## Exchanges

A key element to an effective campaign is the development of attractive exchanges that are motivating and encourage behaviour change^(16,31)^. Campaign messages were placed within the context of family, which the formative research indicated would be particularly attractive^(25)^. Mothers were shown with their children engaged in fun activities that exemplified the healthy changes they were encouraged to incorporate. The campaign conveyed the message that making these small changes would provide opportunities to improve her family’s health and increase family bonding. While some aspects of better health may seem temporally distant (e.g. reducing health risks), family engagement and bonding can have more immediate impacts on family well-being and have been shown to be associated with better diet quality and healthier BMI^(32)^.

## Behaviour change

According to Andreasen, the ultimate objective of social marketing is behaviour change^(16,31)^. The *Be Better* campaign encouraged mothers to make small changes (e.g. add vegetables to favourite recipes, cut FV for snacks and add fruit to water) that could ultimately add up to big improvements in health and well-being. Examples of healthy changes were presented as engaging, simple and doable activities within the context of family, neighbourhood and community. Mothers were directed by the ads to a dedicated website for additional tips and recipes to increase their knowledge, skills and self-efficacy.

## Competition

Competition refers to the behaviours that the audience is accustomed to or prefers as well as to the organisations and companies who promote alternative (sometimes unhealthy) products^(31)^. Families, particularly families of color, are frequently exposed to marketing that promotes unhealthy foods and drinks^(33)^. Not surprisingly, focus group mothers reported that they were influenced by the convenience of fast food and packaged foods – often inexpensive and readily available. Mothers indicated that life was stressful and demanding and that these foods were convenient, inexpensive and provided temporary pleasure. The *Be Better* campaign provided examples and suggestions that were easy, fun, appetising and relatively inexpensive as a counterpoint.

## The marketing mix

### Price

Changing behaviours that one is accustomed to and/or prefers entails a price^(31)^. The price required to improve health entailed the cutting back of convenience foods, fast foods and foods high in added sugar in exchange for short- and longer term improvements in health, mood, cognition and appearance. The cost of healthier foods can at times be more than the cost of less healthy options, thereby increasing the price of making healthy changes. In developing the campaign, substantial effort was made to ensure that the foods, recipes and suggestions promoted by the campaign were nutritionally dense, relatively affordable in California and easy to incorporate.

### The product

In social marketing, the product is the desired behaviour change, the associated benefits and the tangible objects and services that facilitate that change^(31)^. The *Be Better* campaign implied that taking small steps towards increasing FV and water intake and engaging in physical activity could help families live their lives to the fullest. Ads were placed within the context of mothers engaging in simple, fun and easy-to-implement behaviours alongside their families, in keeping with the expressed desire for bonding, for connecting with other mothers and for strategies that were simple, easy and approachable (also noted by other researchers)^(34,35)^. Recipes and suggestions were offered in support of these changes and the dedicated website provided additional information and resources.

### Place

Social marketing must consider the place and time of the desired behaviour as well as the accessibility to the programmes, products and services that support behaviour change^(31)^. The *Be Better* campaign encouraged women to eat healthy, drink water and engage in physical activity that could be carried out in a variety of settings (home, work and neighbourhoods) by themselves, with friends and families and within their community. The dedicated website provided additional tips, ideas and resources.

### Promotion

**Fig. 1:**
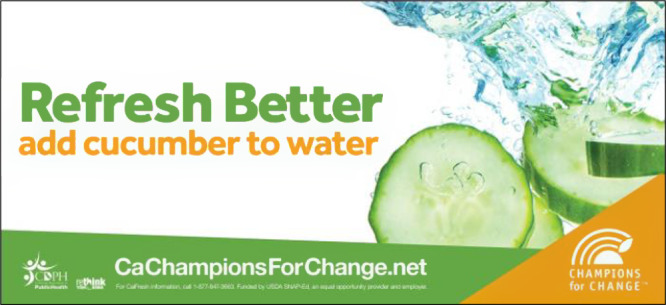
*Be Better* English-Language Billboard Ad

**Fig. 2:**
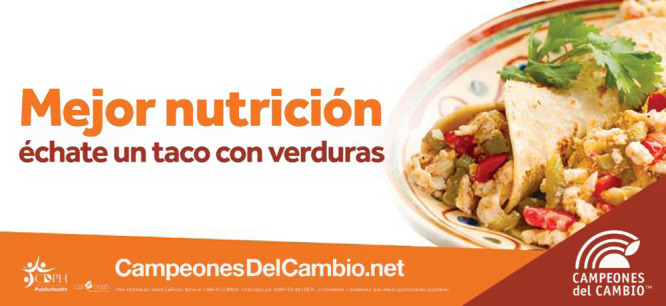
*Be Better* Spanish-Language Billboard Ad

Promotion refers to the messages, materials, channels and activities used to reach the audience^(31)^. The *Be Better* campaign was comprised of English and Spanish language television, radio, digital and outdoor ads (see Figs 1 and 2 examples; *Be Better* television ads can be viewed at https://www.youtube.com/@championsforchange2718/videos, e.g. ‘Champions For Change One,’ ‘Campeones Del Cambio Mas,’ ‘Walk to Feel Better,’ ‘Caminatas’). Each ad directed viewers to the dedicated website (https://calfreshhealthyliving.cdph.ca.gov/en/Pages/default.aspx).

Television, radio and outdoor ads were placed in ten designated market areas (DMA) while digital ads appeared statewide. DMA are geographic areas where residents receive similar local television and radio broadcasts. Four of the ten DMA were designated as evaluation DMA. These evaluation DMA were selected because the primary audience and the placement of campaign ads were well represented. Moreover, these four DMA encompass approximately 84 percent of the total SNAP-Ed eligible population in California. Ad placements were selected to reach at least 51 percent of SNAP-Ed eligible households.

In the following sections, we describe the evaluation of the *Be Better* campaign, with the aim of examining campaign awareness and its association with outcomes of interest. Specifically, we examine associations between mothers’ awareness (recall and recognition) of the ads and the frequency and proportion of mothers’ FV intake, as well as the associations between mothers’ awareness of the ads and several maternal behaviours that may facilitate children’s healthy behaviours.

## Methods

### Design

**Fig. 3:**
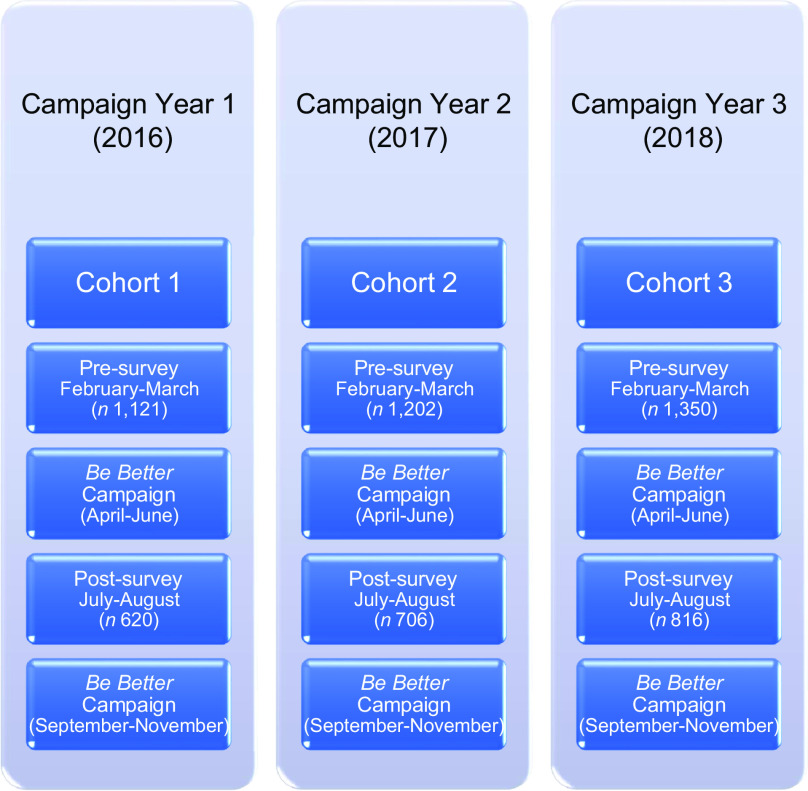
*Be Better* evaluation design

A pre/post evaluation survey was administered to three separate cohorts of SNAP mothers (Fig. 3). A pre-survey (Wave I baseline) was administered approximately 1 month prior to the start of the campaign. The campaign then ran for approximately 3 months. A post-survey (Wave II) was administered a month after the 3-month campaign. The campaign then ran for an additional 3 months (6 months total each year of the campaign). Only those mothers who completed both surveys were included in the analyses.

### Sampling and screening

Households residing within the four evaluation DMA were selected randomly from the California’s Medi-Cal Eligibility Data System (MEDS). The MEDS system is comprised of all individuals who receive SNAP benefits in any given year. Calls were made to the randomly selected households who were invited to participate and screened for eligibility. Participants had to meet the following criteria: Females 18 to 59 years of age, who were mothers or caregivers of one or more children between 5 and 17 years of age, and who self-identified as African American, Latina, White or Asian/Pacific Islander.

### Respondents

Across the 3 years, a total of 3673 mothers participated in Wave I (mean response rate 4 = 14·0 %)^(36)^. Of these, 2229 were re-interviewed at Wave II, for a 60·7 percent average retention rate. Table 1 provides descriptive information for participating mothers at Wave II. For comparison, Table 1 also includes descriptive information on the California SNAP female population who met the criteria for this study and from which the evaluation samples were randomly drawn.

**Table 1:** Demographic characteristics of mothers completing waves I and II and corresponding characteristics of California’s SNAP mothers

Characteristic	Evaluation Sample *n* 2229		Population of California SNAP (Eligible) Mothers*,† (Mean *n* 515 740)	
Race/Ethnicity				
Latina	*n* 890	39·9 %	*n* 264 018	51·3 %
African American	*n* 707	31·7 %	*n* 57 796	11·2 %
White	*n* 588	26·4 %	*n* 85 693	16·6 %
Asian/PI	*n* 44	2·0 %	*n* 4088	0·8 %
Age‡				
Mean Age	37·9 years (range = 18–59)		36·8 years (range = 18–59)	
Survey Language				
English	*n* 1874	84·4 %	*n* 434 107	84·3 %
Spanish	*n* 355	15·6 %	*n* 81 033	15·7 %
Number of Children	m = 1·6		m = 1·9	
Highest Level of Education§				
< High School	*n* 317	14·2 %	Data not available	
High School	*n* 581	26·1 %	Data not available	
Some College	*n* 922	41·4 %	Data not available	
College Graduate	*n* 408	18·3 %	Data not available	

* Represents an estimate (three-year average) of the population of California SNAP mothers eligible to participate in this study from which the evaluation sample was drawn.

† Data source: California Medi-Cal Eligibility Data System (MEDS; 2016–2018). The MEDS system consists of all individuals who receive SNAP benefits (one or more times) during any given year.

‡ One mother did not report level of education.

§ Two mothers did not report age.

Comparisons between the sample and the eligible population of SNAP mothers show similar age, child number and language distributions, but not in race/ethnicity distributions. The sample included a smaller proportion of Latina mothers and a higher proportion of African American and White mothers than seen among the California SNAP population. Other demographic characteristics were not available through the MEDS database.

### Waves I and II interviews

In February (of each evaluation year), a letter of introduction was sent to the randomly selected MEDS households. Telephone calls were then conducted in March, in either English or Spanish. Participants were screened for eligibility and, if eligible, invited to participate in Wave I. The Wave I survey included basic demographic questions, followed by questions on the frequency of mothers’ FV consumption and the proportion of FV consumed. Mothers were asked about behaviours that could facilitate their children’s water and FV consumption and limit their access to unhealthy snacks and SSB. The media campaign then aired from April to June.

Between July and August, participating mothers were administered the Wave II survey, which began with questions assessing mothers’ awareness of the *Be Better* campaign followed by Wave I questions. Mothers were given $10 in appreciation of their time. Study procedures were reviewed and approved by the State of California Committee for the Protection of Human Subjects.

### Assessing unaided recall

Open-ended questions assessed mothers’ recall of the *Be Better* ads. Mothers were asked, ‘I would like to ask you some questions about ads you may have seen or heard. Have you noticed any ads – on television, on the radio, outside on billboards or online – recommending that people be physically active, drink water, or eat fruit and vegetables for better health? I don’t mean ads for specific restaurants or grocery stores’. Those responding ‘yes’ were asked to describe the ads. Interviewers were trained to use probing, open-ended questions to elicit detailed responses. Following the initial question, mothers were asked about ‘any other ads like this in the last three months’. Those answering ‘yes’ were again asked to describe the ads.

### Coding procedure

A catalog of *Be Better* ad contents was developed detailing each campaign ad. Coders used the catalog to assign points to mothers’ ad descriptions. Two coders independently compared mothers’ responses with the elements delineated in the catalog. A third coder trained with the primary coders providing a third, independent judgement in cases where there was disagreement. Based on each mothers’ scores, the points were used to determine whether the mother demonstrated unaided recall. A final determination was made at consensus meetings with all three coders. An analysis of interrater reliability indicated that there was substantial agreement among coders (Cohen’s κ = 0·61; *P* < 0·001).

The unaided recall measure likely underestimates the actual rate of recall. Mothers had to quickly recollect specific and substantial details of one or more ads and articulate those details clearly to the interviewer – a rather difficult task. The aided-recall (recognition) questions that followed complement and supplement the recall task as a measure ad awareness.

### Assessing aided recall (recognition)

The interviewers presented mothers with narratives of campaign ads. After each description, mothers were asked, ‘Do you remember hearing/seeing this ad?’ Mothers who responded ‘yes’ to any of the ad narratives were coded as demonstrating ‘aided recall’. Mothers who responded ‘no’ to all narratives were coded as demonstrating ‘no recall’.

The number of ads recognised served as a proxy of ad exposure. It was assumed that the number of different ads recognised corresponded with the degree of campaign exposure. Mothers who recognised five different ads, for example, were assumed to have had more exposure to the media campaign than mothers who recognised fewer ads. Decoy ad narratives were included and utilised as a statistical control of response bias (i.e. tendency to say ‘yes’) to partially account for biases in memory and responding.

### Analytic plan

Changes in outcomes were examined using generalised estimating equation^(37)^ models, which provide population-level estimates. Generalised estimating equation analyses are frequently utilised for repeated measures designs that test hypotheses using binary (e.g. yes/no) or discrete data (e.g. number of FV consumed). Generalised estimating equation modeling allows for more confidence in statistical conclusions because it produces parameter estimates that are more efficient and unbiased than ordinary least squares regression analyses.

Analyses began with an examination of changes over time (with participants nested within campaign year), without the ad awareness variables included to determine how mothers’ behaviours changed over the survey period, irrespective of the effect of the ads. Each outcome variable (e.g. FV consumption) was entered as a dependent variable, with survey periods as a repeated measure, and race/ethnicity, education, interview language and age as predictor variables.

Following the initial analyses, the ad awareness variables (recall, recognition and response bias) were entered into the models to examine the unique contribution of the campaign. Finally, we also examined ad recognition as a function of media channel (i.e. television, billboards, radio and digital) for a more fine-grained analysis of recognition.

## Results

### Campaign recall and recognition

A total 446 mothers (20·0 %) were categorised as demonstrating unaided recall, 1378 (61·8 %) as demonstrating aided recall and 405 (18·2 %) as showing no recall of the ads. White mothers (28·3 %) were more likely to be categorised as no-recall mothers than Latina (13·6 %) and African American mothers (14·6 %; X^2^ = 87·2, adjusted *P* < 0·001). Mothers with less than a high school education (11·0 %) were also less likely to be categorised as no-recall mothers than mothers with higher educational backgrounds (≥ 18·1 %; X^2^ ≥ 15·3, adjusted *ps* = 0·02).

Because the number of ads differed from year to year, we report recognition as a percentage of recognised ads. The mean percentage of ads recognised overall was 21·6 %. White mothers (28·1 %) recognised a smaller percentage of ads than Latinas (46·0 %) and African Americans (43 %; F = 19·5, Bonferroni adjusted *ps* < 0·001), but not Asian/Pacific Islander (PI) mothers (22·4 %, *P* > 0·05). Spanish-speaking mothers (59·0 %) recognised more ads than English-speaking mothers (37·0 %; F = 70·6, adjusted *P* < 0·001). One explanation for the higher rate among Spanish-speaking mothers may be that some mothers were bilingual and may have been exposed to both English- and Spanish-language ads, thereby increasing their overall exposure.

Mothers with less than a high school education (51·0 %) recognised more ads than did mothers with higher educational backgrounds (41·0 % or less; Fs ≥ 8·8, adjusted *ps* < 0·001).

**Fig. 4:**
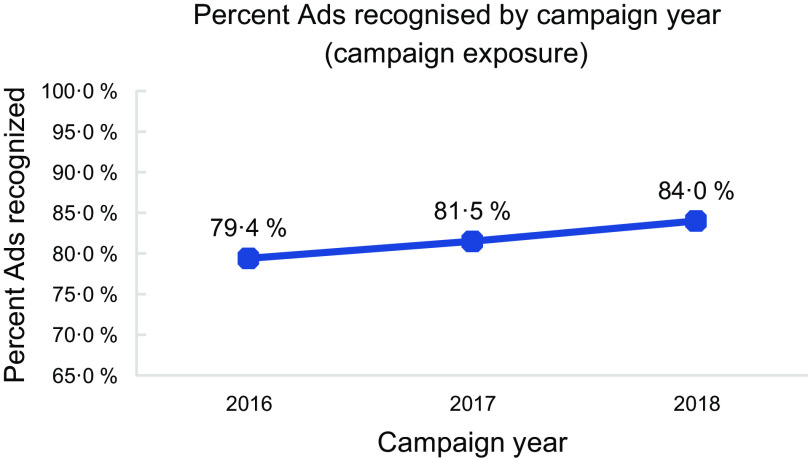
Percent of Ads recognised by campaign year

On average, 82 percent of surveyed mothers demonstrated awareness (recall/recognition) of the campaign across the three campaign years, increasing steadily and significantly each year: 79·4 % in 2016, 81·5 % in 2017 and 84·0 % in 2018 (Fig. 4; F = 124·4, *P* < 0·001).

### Behavioural outcomes

We first present changes in response patterns (if any) that occurred between Waves *without* the inclusion of the ad awareness variables to examine potential behavioural changes not specific to the campaign. These analyses were followed with analyses that *included* the ad awareness variables to examine the unique contribution of the campaign. We then examine each outcome as a function of media channel. Only statistically significant associations are reported for brevity.

It was expected that mothers who were exposed to the campaign, particularly as mothers’ campaign exposure increased, would show evidence of positive changes.

### Maternal fruit and vegetable consumption

This variable was computed from responses to five questions: Mothers were asked about the number of times (per day, week or month) they drank 100 percent fruit juice; ate fresh, frozen, canned or dried fruit; ate green salad; ate carrots and ate other vegetables. Responses were converted to *times per day* and then summed across the five categories, representing the number of times (or frequency) FV were consumed each day.

**Fig. 5:**
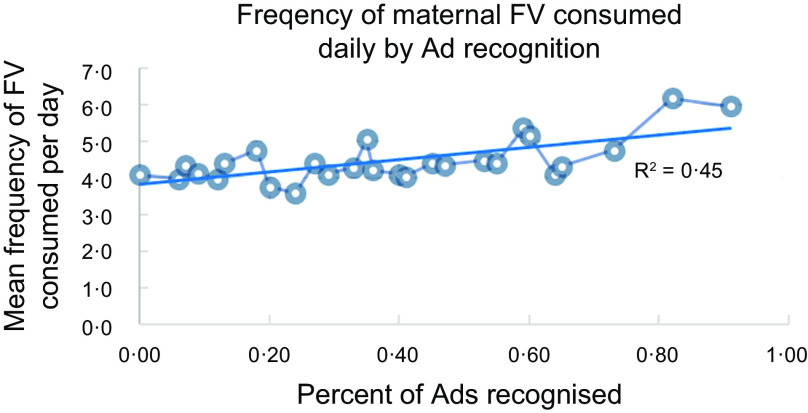
Frequency of maternal FV consumed daily by Ad recognition. FV, fruit and vegetable

There was a significant positive association between ad recognition and FV consumption. As the number of recognised ads increased, daily FV consumption also increased (*β* = 0·19; Z = 3·02, *P* = 0·002 (Fig. 5). For each sd (0·20) above the average number of ads recognised (m = 2·3 ads), the frequency of FV consumption increased by 0·44 times per day – almost a one-half time increase in FV frequency.

Latina (m = 4·3, std. = 3·0; *β* = 0·12) and African American (m = 4·6, std. = 3·2; *β* = 0·16) mothers consumed FV more frequently than did White mothers (m = 3·9, std. = 2·5; Zs ≥ 3·08, *ps* ≤ 0·002). Asian/PI mothers (m = 4·2, std. = 2·6) did not differ significantly from other mothers (*P* > 0·05).

### Media channels

As the percentage of recognised billboard ads increased, the frequency of FV consumption also increased (*β* = 0·20; Z = 3·85, *P* = 0·001) in campaign years 2017 (*β* = 0·28; Z = 2·09, *P* = 0·04) and 2018 (*β* = 0·19; Z = 3·59, *P* < 0·001). This pattern was also observed for digital ads, but only in 2018 (*β* = 0·17; Z = 2·75, *P* = 0·01).

### Proportion of plate containing fruit and vegetables

Mothers were asked: ‘How much of your plate is usually filled with fruit and vegetables? Would you say none, one fourth, one half, three fourths, or all?’ Responses were dichotomised as either meeting (half or more FV on a plate) or not meeting (< half) the USDA FV recommendation.

A significant recognition × campaign year interaction emerged. As ad recognition increased, the likelihood of meeting the recommendation also increased, but only in year 2017 (*β* = 0·73; Z = 3·74, *P* < 0·001). For each sd increase in ads recognised, there was an increase of 1·6 mothers who reported consuming at least half plate of FV.

Mothers with less than high school education (m = 0·37, std. = 0·48; *β* = –0·04) or with a high school degree (m = 0·41, std. = 0·49; *β* = –0.05) were less likely to meet the USDA recommendation than mothers with higher education levels (ms ≥ 0·44, std. = 0·50; Zs ≥ 2·55; *ps* ≤ 0·04). In general, as maternal age increased, the more likely mothers were to meet the USDA recommendation (*β* = 0·01; Z = 2·56, *P* = 0·01).

### Media channels

As the percentage of recognised television ads increased, the likelihood of meeting the USDA recommendation also increased, but only in 2018 (*β* = 0·19; Z = 2·26, *P* = 0·02). A similar pattern was observed for billboards (*β* = 0·28; Z = 3·28, *P* = 0·001) and digital ads in 2017 (*β* = 0·11; Z = 3·19, *P* = 0·001).

### Children’s fruit and vegetable consumption

Mothers were asked: ‘How often do you make it easy for (your/any of your) child/ren living in your home to eat fruit and vegetables, such as by having them washed, cut and ready to eat? Would you say not at all, less than every month, every month, every week, or every day?’ Mothers who indicated they made it easy for their children to eat FV daily were coded as FV facilitative.

In general, a greater proportion of mothers were categorised as FV facilitative at Wave II (m = 0·73, std. = 0·46; *β* = 0·03) than at Wave I (m = 0·71; std. = 0·64; Z = 2·09, *P* = 0·04).

A significant main effect of recognition emerged. As the number of recognised ads increased, the number of mothers who were categorised as FV facilitative also increased (*β* = 0·16; Z = 2·13, *P* = 0·03). For each sd increase in ads recognised, there was an increase of 0·48 mothers who were categorised as facilitating their children’s daily FV consumption.

Spanish-speaking mothers (m = 0·74, std. = 0·44; *β* = 0·15) were more likely to be categorised as FV facilitative than were English-speaking mothers (m = 0·71, std. = 0·45; Z = 3·78, *P* < 0·001).

Latina (m = 0·72, std. = 0·45; *β* = –0·07) and African American mothers (m = 0·67, std. = 0·47; *β* = –0·12) were less likely to be categorised as FV facilitative than were White mothers (m = 0·76, std. = 0·43; Zs ≥ 2·24, *ps* ≤ 0·03), but did not differ from Asian/PI mothers (m = 0·69, std. = 0·47; *ps* < 0·05).

Mothers with a high school education (m = 0·67, std. = 47; *β* = –0·21) or less (m = 0·68, std. = 0·47; *β* = –0·15) were less likely to be categorised as FV facilitative than mothers with some college education (m = 0·73, std. = 44) or a college degree (m = 0·77; std. = 0·42; Zs ≥ 4·24, *ps* ≤ 0·001).

### Media channels

As the number of recognised billboard ads increased, mothers were more likely to be categorised as FV facilitative (*β* = 0·12; Z = 2·36, *P* = 0·02). A similar pattern emerged for digital ads, but only in 2017 (*β* = 0·13; Z = 2·21, *P* = 0·03).

### Limits on children’s unhealthy snacks

Mothers were asked: ‘Have you set limits on the amount of “unhealthy food” you have in the home for your child or children to snack on?’ Responses were recorded as either ‘yes’ or ‘no’. Mothers who responded ‘yes’ were coded as unhealthy snack limiting.

There was a positive association between recognition and limits on unhealthy snacks. The greater the number of ads recognised, the greater the likelihood that mothers limited their children’s unhealthy snacks (*β* = 0·11; Z = 3·25, *P* = 0·001). For each sd increase in ads recognised, there was an 0·20 increase in the number of mothers who limited unhealthy snacks.

Mothers with a high school education (m = 0·91, std. = 0·29; *β* = –0·05) or less (m = 0·88, std. = 0·32; *β* = –0·08; Zs ≥ 3·47, *ps* = 0·001) were less likely to limit unhealthy snacks than mothers with some college education (m = 0·93, std. = 0·26) or a college degree (m = 0·94, std. = 0·23; Zs ≤ 1·94, *ps* ≥ 0·05).

Generally, the older the mother, the less likely they were to limit their children’s unhealthy snacks (*β* = –0·01; Z = 3·33, *P* = 0·001).

### Media channels

The greater the number of television ads recognised, the more likely mothers were to limit children’s unhealthy snacks but only in 2018 (*β* = 0·05; Z = 2·97, *P* = 0·003). There was also a marginally significant interaction in year 2017 (*β* = 0·03; Z = 1·85, *P* = 0·07). A similar pattern emerged for billboard ads (*β* = 0·06; Z = 3·00, *P* = 0·003) and digital ads (*β* = 0·08; Z = 3·74, *P* < 0·001) in campaign year 2018 (*β* = 0·08; Z = 3·74, *P* < 0·001).

### Children’s water consumption

Mothers were asked, ‘How often do you make it easy for your child or the children living in your home to drink water, such as by having a filled pitcher or bottles of water available? Would you say not at all, less than every month, every month, every week, or every day?’ Mothers who made it easy for their children to drink water daily were coded as water facilitative.

Latina (m = 0·98, std. = 0·15; *β* = –0·02) and African American mothers (m = 0·98, std. = 0·15; *β* = –0·04) were less likely to be categorised as water facilitative than were White mothers (m = 0·99, std. = 0·09; Zs ≥ 2·54, *ps* ≤ 0·01), but not compared with Asian/PI mothers (m = 0·99, std. = 0·05, *P* > 0·05).

No main effects of ad awareness were observed, but some effects emerged for media channels.

### Media channels

The greater the number of television ads recognised, the more likely mothers were to be categorised as water facilitative in year 2018 (*β* = 0·02; Z = 2·29, *P* = 0·04). A similar pattern emerged for radio ads in 2016 (*β* = 0·02; Z = 2·15, *P* = 0·03), with a marginally significant effect in 2018 (*β* = 0·02; Z = 1·86, *P* = 0·07).

### Limits on children’s sugar-sweetened beverage consumption

Mothers were asked: ‘Have you set limits on the amount of sweetened beverages you have in the home for children to drink? By sweetened beverages, I mean soda, sports drinks, and sugar sweetened juice drinks’. Responses were recorded as either ‘yes’ or ‘no’. Mothers who responded ‘yes’ were coded as SSB limiting.

The proportion of mothers who indicated they set limits on their children’s SSB consumption increased from Wave I (m = 0·93, std. = 0·25; *β* = 0·02) to Wave II (m = 0·95, std. = 0·22; Z = 2·46, *P* = 0·01).

Unaided recall mothers (m = 0·95, std. = 0·22; *β* = 0·03) were more likely to limit their children’s SSB consumption than no-recall mothers (m = 0·93, std. = 0·93, std. = 0·26; Z = 2·20, *P* = 0·03). There was also a significant main effect of recognition. The greater the number of ads recognised, the more likely mothers were to limit children’s SSB (*β* = 0·07; Z = 3·52, *P* < 0·001). For each sd increase in ad recognition, there was a 0·16 increase in the number of mothers who limited access to SSB.

African American mothers (m = 0·93, std. = 0·26; *β* = –0·03) were less likely to limit SSB than White mothers (m = 0·95, std. = 0·22; Z = 2·51, *P* = 0·01), but did not differ from Latina mothers (m = 0·94; std. = 0·22, *P* > 0·05) or Asian/PI mothers (m = 0·94, std. = 0·27; *ps* > 0·05).

Mothers with less than a high school education (m = 0·92, std. = 0·28; *β* = –0·04) were less likely to limit children’s SSB than mothers with some college education (m = 0·95, std. = 0·22) or a college degree (m = 0·95, std. = 22; Z = 2·80, *P* = 0·01). Generally, the older the mother, the more likely they were to limit children’s SSB (*β* = –0·01; Z = 4·75, *P* < 0·001).

### Media channels

The greater the number of television ads recognised, the more likely mothers were to limit SSB consumption, but only in campaign year 2018 (*β* = 0·04; Z = 2·68, *P* = 0·01). Similar patterns were seen for billboard ads (*β* = 0·04; Z = 2·37, *P* = 0·02), radio ads (*β* = 0·03; Z = 3·77, *P* < 0·001) and digital ads (*β* = 0·04; Z = 2·68, *P* = 0·01) in campaign year 2018 (*β* = 0·05; Z = 3·10, *P* = 0·002).

## Discussion

The SNAP-Ed program reaches over 90 million Americans with low incomes in the USA through its programmes and interventions^(38)^. An increasing number of SNAP-Ed implementing agencies have included (or plan to include) a social marketing campaign^(39)^. The number of published social marketing projects that focus on SNAP-Ed populations remains relatively limited in number^(28–30,34,35,40)^,with most focusing on FV consumption in adults^(39)^. The present study contributes to this literature with a multi-year campaign based on Andreasen’s benchmark criteria, including an evaluation utilising population-based analyses.

The CDPH, with its creative partners, developed the *Be Better* campaign to encourage California SNAP-Ed mothers to increase the frequency and proportion of FV consumed and to increase behaviours that facilitate children’s healthy behaviours. Campaign awareness steadily increased over 3 years; a trend also observed in the *Healthy Choices Catch On* campaign, which has aired for several years^(30)^. The *Catch On* campaign found that adults exposed to campaign more than ten times consumed more FV than those exposed fewer times. Similarly, we found that the more campaign ads mothers recognised (indicative of greater exposure), the more likely mothers were to report increased FV consumption and portion sizes, to facilitate their children’s FV consumption, and to limit their children’s unhealthy snacks and SSB. Relatively long campaign time frames, therefore, may be necessary to achieve high campaign reach, brand recognition and measurable behaviour changes^(29,34,41,42)^.

Associations between ad awareness and outcomes varied by year. Although the campaign included FV, water, and physical activity ads, the focus of these ads varied by year, and the outcomes seem to reflect these variations in yearly focus. In 2016, the ads were heavily weighted towards physical activity (not discussed here), with fewer numbers of FV and water ads. In 2017, a greater number of FV and water ads were included, and in 2018, the number of water ads increased substantially. Reflecting these variations in focus, significant associations were observed between ad awareness and FV/Snacks in 2017 and with water and SSB in 2018. Though the campaign aired for 6 months each year, the associations between ad awareness and outcomes were evident primarily in 2017 and 2018, suggesting that content specific and repeated messaging may be necessary for effects to emerge. Findings by media channel also indicated that utilisation of a wide range of media channels (that also take into consideration varied viewing preferences)^(35)^ may be necessary to increase campaign reach and impact.

Consistent with mothers’ desire to bond with their children, and with the expectation that mothers influence their family’s health behaviours, the *Be Better* messages included children (as the downstream audience)^(43)^ and families in campaign ads. Research indicates that there is a positive relationship between children’s FV consumption and parental behaviours, with food access and availability being strong determinants of FV consumption among adolescents and children^(44)^. Messages that model healthy parent behaviours and suggest ways to increase healthy food access (e.g. cut FV as snacks, have a water pitcher in the refrigerator) can have a positive influence on children’s health behaviours.

Ad awareness was generally not associated with mothers’ support for children’s water consumption, though media channel analyses indicated that television and radio ad recognition was associated with children’s increased water consumption as reported by the parent. It is possible that television and radio ads were viewed more often than other types of media channels or that these ads were more engaging than static digital or outdoor ads. It also may reflect the limited proportion of water promoting ads during the first, and to a lesser extent, during the second year.

Few social marketing studies have included water promotion in their campaigns. One such campaign was the 5–4–3–2–1 Go! project^(45)^. Parents exposed to campaign messages reported drinking more water than those who were not exposed. In addition to campaign exposure, parents were given ‘refrigerator magnets, water bottles and other items that may have served as shopping reminders’. The authors suggested that these items may have also contributed to increased water consumption. It is unclear from these findings what aspects of the campaign accounted for the increase in adult water consumption. Additional examples of social marketing campaigns promoting water consumption would be helpful in determining the types of messages and media channels that could increase water consumption among adults and children.

A mothers’ race/ethnicity was found to be associated with behaviours that facilitate their children’s water intake. White mothers were more likely to report facilitating their children’s water intake than Latina and African American mothers. Data from national samples show that non-Hispanic White adults tend to consume more water overall and drink more tap water than non-Hispanic Black and Mexican American adults who consume the least amount of water generally and who drink the least amount of tap water^(46)^. Latino and African American adults are more likely to perceive tap water as unsafe than White adults, which may account in part for some of the race/ethnicity differences in water consumption^(46)^. Similar to the income-related differences in eating patterns, adults with higher incomes are more likely to consume tap water than adults with lower incomes^(47)^. It is possible that higher income individuals tend to live in areas where water is more likely to be (or perceived to be) safe to drink. Increasing water consumption through social marketing may necessitate addressing issues of actual and/or perceived water safety.

Ad awareness was also associated with mothers’ educational attainment. Mothers with lower educational attainments (e.g. less than high school) were less likely to be categorised as demonstrating unaided recall than other mothers. This difference was not evident with recognition. Providing a sufficiently detailed verbal recall narrative is a difficult task relative to recognition where a simple yes/no response is sufficient. Educational experience, rather than memory per se, may limit measures of recall in social marketing evaluations^(48,49)^. Including a varied set of ad awareness measures (e.g. recall, confirmed recall and recognition) may thus be necessary to detect campaign reach with greater precision and lessen the possible confounding effects of educational experiences.

### Limitations

The final sample of randomly surveyed mothers was similar in age, number of children, and language distributions as the SNAP population from which they were drawn, though differences in race/ethnicity proportions arose. Demographic characteristics available through the California MEDS database were limited to age, gender, language, number of children in household, and race/ethnicity, which precluded our ability to examine the results, and its generalisability, along other potentially important characteristics. Moreover, the present study relied on self-report rather than objective measures of behaviour, which may reduce the accuracy/precision of the outcome measures.

Unaided recall is a key measure in SNAP-Ed social marketing campaigns, yet few researchers operationalise recall, making comparisons difficult to assess. Including a description of how recall is defined and measured would improve comparability across studies. Researchers have generally relied on cross-sectional, pre/post or post-only designs to evaluate campaigns, limiting our understanding of the longer-term effectiveness of social marketing projects. It is recommended that future campaigns be evaluated using longitudinal designs that could allow examination of the ebbs and flows of campaign effectiveness over time. Furthermore, an increasing number of social marketing researchers are moving toward a systems approach that addresses the complex environments influencing behaviour^(23,50)^. To the best of our knowledge, there are currently no published studies of social marketing campaigns (focused on SNAP-Ed populations) that have taken a systems approach to social marketing and evaluation.

## Conclusions

The *Be Better* campaign illustrates how Andreasen’s social marketing benchmarks can be utilised to develop an effective campaign. The *Be Better* campaign successfully reached its intended audience of diverse SNAP-Ed California mothers. Campaign awareness increased steadily over time and as expected was associated with increases in FV consumption and portion size, as well as increases in the number of mothers who engaged in behaviours supportive of their children’s healthy behaviours. Associations varied by message focus, year and media channel, highlighting the importance of content-specific messaging, extended media exposure and the use of a wide range of media channels for campaign effectiveness. Though effects sizes varied from small to large, the potential reach of social marketing campaigns makes it a powerful tool for health change among SNAP-Ed families.
